# Supplementary figures and images for: Impact of sampling depth on pathogen detection in pit latrines
Source: PLoS Negl Trop Dis. 2021 Mar 2;15(3):e0009176. doi: 10.1371/journal.pntd.0009176 (PMC7954291; doi:10.1371/journal.pntd.0009176)

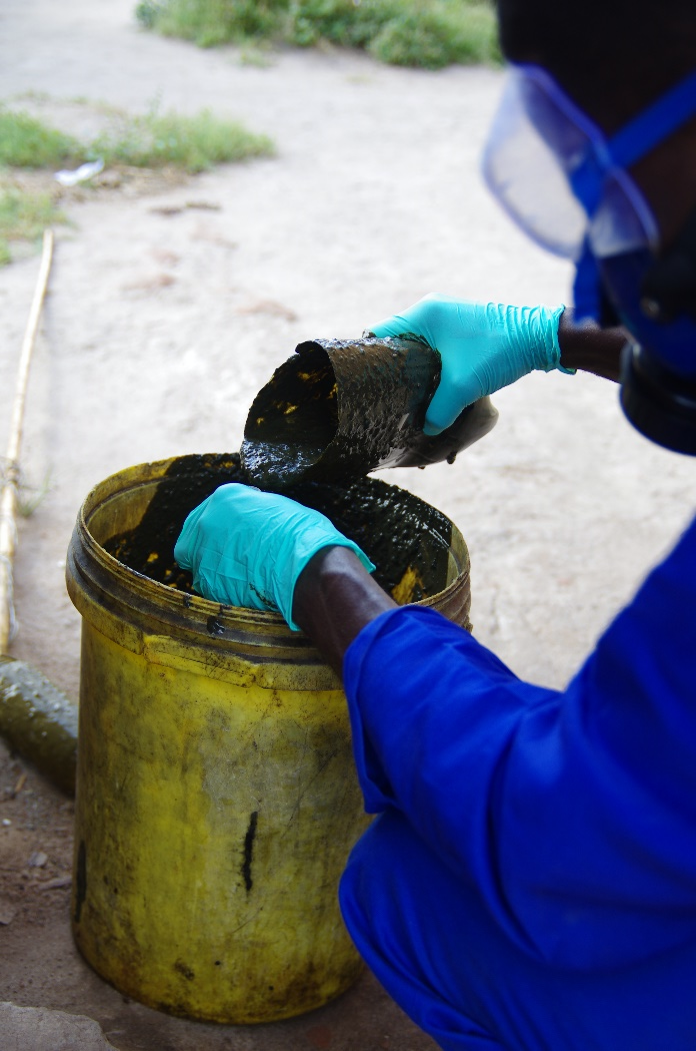

Supplement: S1 Fig — (TIF) [file pntd.0009176.s001.tif]

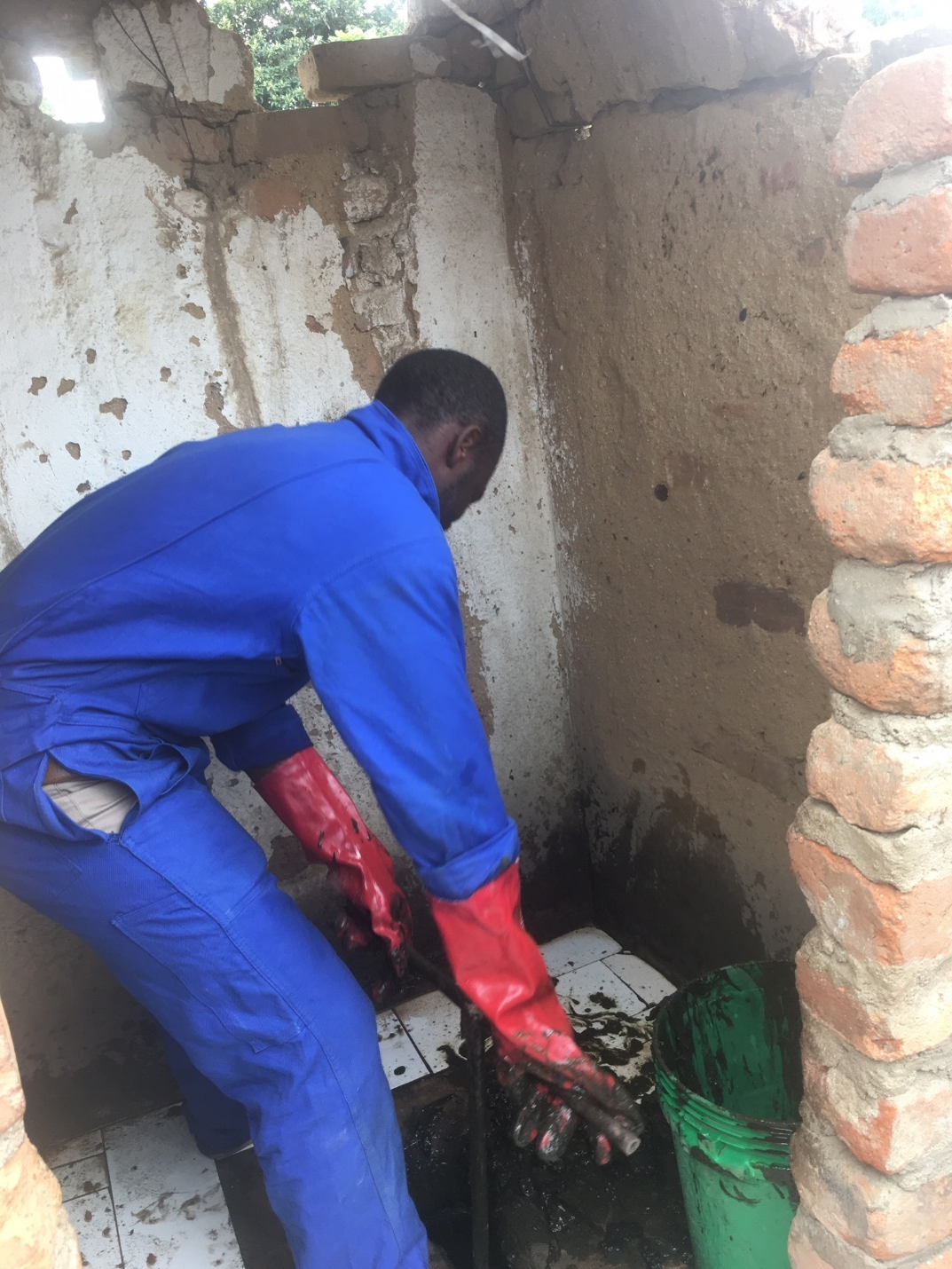

Supplement: S2 Fig — (TIF) [file pntd.0009176.s002.tif]

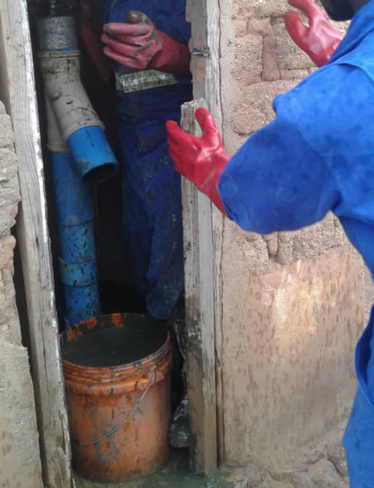

Supplement: S3 Fig — (TIF) [file pntd.0009176.s003.tif]

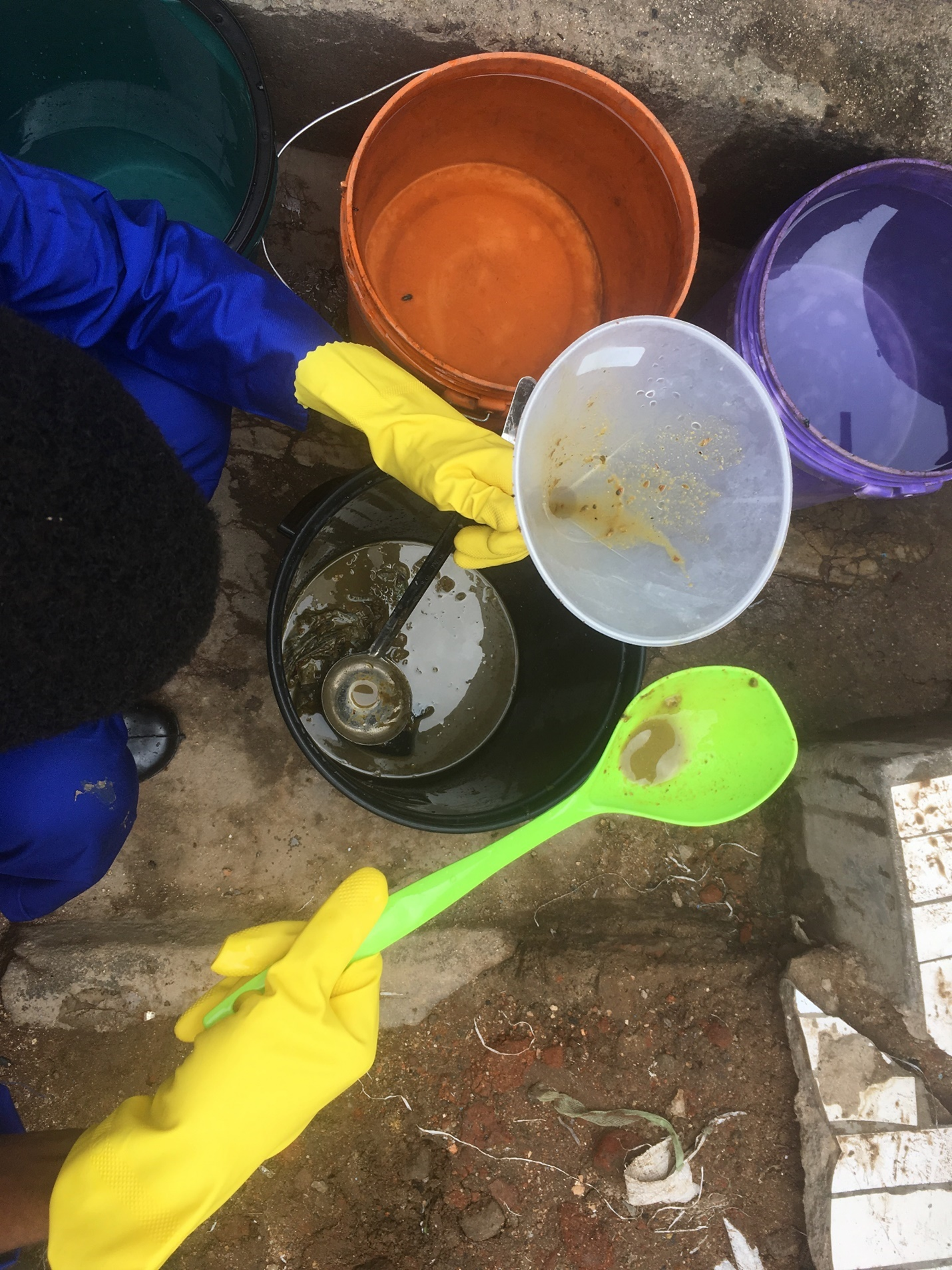

Supplement: S4 Fig — (TIF) [file pntd.0009176.s004.tif]
