# Supplementary material for: Impact of sampling depth on pathogen detection in pit latrines
Source: PLoS Negl Trop Dis. 2021 Mar 2;15(3):e0009176. doi: 10.1371/journal.pntd.0009176 (PMC7954291; doi:10.1371/journal.pntd.0009176)
Supplement: S1 Text — (DOCX) [file pntd.0009176.s005.docx]

Text S1. Methodology for total nucleic acid extraction from fecal sludges.

Fecal sludges arrived at Georgia Institute of Technology, Atlanta, USA preserved in UNEX buffer. We stored samples at -80^o^ Celsius until analysis. After thawing, we vortexed each fecal sludge sample for 3 seconds, then pipetted 100 μL of the sludge-UNEX buffer mixture from the bottom of the sample tube into a Bertin SK-38 (Bertin Corp, Rockville, MD) bead beating tube with 1 mL of Qiagen Buffer ASL (Qiagen, Hilden, Germany). We spiked in phage MS2 as an extraction control. We vortexed bead beating tubes for 5 minutes, incubated at room temperature for 15 minutes and then centrifuged at 14,000 rpm for 2 minutes. We proceeded with extraction following the manufacturer’s protocol for the QIAamp 96 Virus QIAcube HT Kit, which we automated on the QIAcube (Qiagen, Hilden, Germany).
