# Supplementary material for: Impact of sampling depth on pathogen detection in pit latrines
Source: PLoS Negl Trop Dis. 2021 Mar 2;15(3):e0009176. doi: 10.1371/journal.pntd.0009176 (PMC7954291; doi:10.1371/journal.pntd.0009176)
Supplement: S2 Text — (DOCX) [file pntd.0009176.s006.docx]

Text S2. Custom TaqMan Array Card (TAC)

We describe the TAC platform in detail in Capone *et al*. 2020. Briefly, TAC is a 384-well array card with 8 ports for loading samples and each 1-µL well contains dried-down primers and hydrolysis probes for the detection of defined targets. We purchased custom TACs produced by Thermo Fisher Scientific (Waltham, MA) using the primers and probes listed in Table S1.

For analysis, we mixed 50 μL of total nucleic acid template (0.5 μL template per reaction well) with 50 μL of qScript XLT 1-Step RT-qPCR ToughMix (Quantabio, Beverly, MA), then filled ports 2-7 with the combined 100 μL. In total we tested 6 samples per card, using the first port as a negative control and the last port as a positive control, for which we used individual aliquots of our combined positive control material (gene targets inserted into plasmids) (IDT, Coralville, IA). Combined positive controls were developed using methods from Kodani *et al.* 2012 (PMID: 22170926). Following the manufacturer’s instructions, we centrifuged each card twice at 1,200 rpm for one minute, sealed the card, trimmed the loading ports, and loaded the card into a QuantStudio 7 (Thermo Fisher Scientific, Waltham, MA). To perform reverse transcription real-time PCR we used the following cycling conditions with a 1̊ C/s ramp rate between all steps: 45̊ C for 10 minutes, 94̊ C for 10 minutes, and then 45 cycles of 94̊ C for 30 seconds and 60̊ C for 1 minute. All positive controls amplified as expected (typically ~ Ct = 28-30 depending on the assay) and we detected MS2 in all samples. In addition, the TAC included an internal positive control (TaqMan™ Exogenous Internal Positive Control, Applied Biosystems, Foster City, CA), which we used to monitor for potential inhibition. The internal positive control assay amplified consistently with no indication of inhibition (average Ct = 25, range =24-26). We observed positive amplification for all assays using our positive controls (n = 13). We observed no amplification for any assay in any of our extraction controls (n=5) or no template controls (n=8) below a quantification cycle (Cq) of 40.
