# Supplementary material for: Impact of sampling depth on pathogen detection in pit latrines
Source: PLoS Negl Trop Dis. 2021 Mar 2;15(3):e0009176. doi: 10.1371/journal.pntd.0009176 (PMC7954291; doi:10.1371/journal.pntd.0009176)
Supplement: S1 Table — (DOCX) [file pntd.0009176.s007.docx]

Table S1. Assays used on the custom TAC.

| Target | Assay reference |
| --- | --- |
| **Bacteria** |  |
| *Campylobacter coli* | Cunningham, S. A.; Sloan, L. M.; Nyre, L. M.; Vetter, E. A.; Mandrekar, J.; Patel, R. Three-Hour Molecular Detection of Campylobacter, Salmonella, Yersinia, and Shigella Species in Feces with Accuracy as High as That of Culture Downloaded From. *J. Clin. Microbiol.* **2010**, *48* (8), 2929–2933. |
| *Campylobacter jejuni* | Cunningham, S. A.; Sloan, L. M.; Nyre, L. M.; Vetter, E. A.; Mandrekar, J.; Patel, R. Three-Hour Molecular Detection of Campylobacter, Salmonella, Yersinia, and Shigella Species in Feces with Accuracy as High as That of Culture Downloaded From. *J. Clin. Microbiol.* **2010**, *48* (8), 2929–2933. |
| *Clostridium difficile* (*tcdA*) | Houser, B. A.; Hattel, A. L.; Jayarao, B. M. Real-Time Multiplex Polymerase Chain Reaction Assay for Rapid Detection of Clostridium Difficile Toxin-Encoding Strains. *Foodborne Pathog. Dis.* **2010**, *7* (6), 719–726. |
| *Clostridium difficile* (*tcdB*) | Houser, B. A.; Hattel, A. L.; Jayarao, B. M. Real-Time Multiplex Polymerase Chain Reaction Assay for Rapid Detection of Clostridium Difficile Toxin-Encoding Strains. *Foodborne Pathog. Dis.* **2010**, *7* (6), 719–726. |
| *E. coli / Shigella* (*ipaH* gene) | Thiem, V. D.; Sethabutr, O.; Seidlein, L. von; Tung, T. Van; Canh, D. G.; Chien, B. T.; Tho, L. H.; Lee, H.; Houng, H.-S.; Hale, T. L.; et al. Detection of Shigella by a PCR Assay Targeting the IpaH Gene Suggests Increased Prevalence of Shigellosis in Nha Trang, Vietnam. *J. Clin. Microbiol.* **2004**, *42* (5), 2031–2035. |
| EAEC (*aaiC* gene) | Liu, J.; Gratz, J.; Amour, C.; Kibiki, G.; Becker, S.; Janaki, L.; Verweij, J. J.; Taniuchi, M.; Sobuz, S. U.; Haque, R.; et al. A Laboratory-Developed Taqman Array Card for Simultaneous Detection of 19 Enteropathogens. *J. Clin. Microbiol.* **2013**, *51* (2), 472–480. |
| EAEC (*aatA* gene) | Boisen, N.; Struve, C.; Scheutz, F.; Krogfelt, K. A.; Nataro, J. P. New Adhesin of Enteroaggregative Escherichia Coli Related to the Afa/Dr/AAF Family. *Infect. Immun.* **2008**, *76* (7), 3281–3292. |
| EPEC (*bfpA* gene) | Liu, J.; Gratz, J.; Amour, C.; Kibiki, G.; Becker, S.; Janaki, L.; Verweij, J. J.; Taniuchi, M.; Sobuz, S. U.; Haque, R.; et al. A Laboratory-Developed Taqman Array Card for Simultaneous Detection of 19 Enteropathogens. *J. Clin. Microbiol.* **2013**, *51* (2), 472–480. |
| EPEC (*eae* gene) | Liu, J.; Gratz, J.; Amour, C.; Kibiki, G.; Becker, S.; Janaki, L.; Verweij, J. J.; Taniuchi, M.; Sobuz, S. U.; Haque, R.; et al. A Laboratory-Developed Taqman Array Card for Simultaneous Detection of 19 Enteropathogens. *J. Clin. Microbiol.* **2013**, *51* (2), 472–480. |
| ETEC-LT | Hidaka, A.; Hokyo, T.; Arikawa, K.; Fujihara, S.; Ogasawara, J.; Hase, A.; Hara-Kudo, Y.; Nishikawa, Y. Multiplex Real-Time PCR for Exhaustive Detection of Diarrhoeagenic *Escherichia Coli*. *J. Appl. Microbiol.* **2009**, *106* (2), 410–420. |
| ETEC-ST | Liu, J.; Gratz, J.; Amour, C.; Kibiki, G.; Becker, S.; Janaki, L.; Verweij, J. J.; Taniuchi, M.; Sobuz, S. U.; Haque, R.; et al. A Laboratory-Developed Taqman Array Card for Simultaneous Detection of 19 Enteropathogens. *J. Clin. Microbiol.* **2013**, *51* (2), 472–480. |
| *Salmonella* | Liu, J.; Gratz, J.; Amour, C.; Kibiki, G.; Becker, S.; Janaki, L.; Verweij, J. J.; Taniuchi, M.; Sobuz, S. U.; Haque, R.; et al. A Laboratory-Developed Taqman Array Card for Simultaneous Detection of 19 Enteropathogens. *J. Clin. Microbiol.* **2013**, *51* (2), 472–480. |
| Shiga-like toxin 1 (*stx1*) | Liu, J.; Gratz, J.; Amour, C.; Kibiki, G.; Becker, S.; Janaki, L.; Verweij, J. J.; Taniuchi, M.; Sobuz, S. U.; Haque, R.; et al. A Laboratory-Developed Taqman Array Card for Simultaneous Detection of 19 Enteropathogens. *J. Clin. Microbiol.* **2013**, *51* (2), 472–480. |
| Shiga-like toxin 2 (*stx2*) | Hidaka, A.; Hokyo, T.; Arikawa, K.; Fujihara, S.; Ogasawara, J.; Hase, A.; Hara-Kudo, Y.; Nishikawa, Y. Multiplex Real-Time PCR for Exhaustive Detection of Diarrhoeagenic *Escherichia Coli*. *J. Appl. Microbiol.* **2009**, *106* (2), 410–420. |
| *Vibrio cholerae* | Liu, J.; Gratz, J.; Amour, C.; Kibiki, G.; Becker, S.; Janaki, L.; Verweij, J. J.; Taniuchi, M.; Sobuz, S. U.; Haque, R.; et al. A Laboratory-Developed Taqman Array Card for Simultaneous Detection of 19 Enteropathogens. *J. Clin. Microbiol.* **2013**, *51* (2), 472–480. |
| *Yersinia* spp. | Liu, J.; Gratz, J.; Maro, A.; Kumburu, H.; Kibiki, G.; Taniuchi, M.; Howlader, A. M.; Sobuz, S. U.; Haque, R.; Talukder, K. A.; et al. Simultaneous Detection of Six Diarrhea-Causing Bacterial Pathogens with an In-House PCR-Luminex Assay. *J. Clin. Microbiol.* **2012**, *50* (1), 98–103. |
| **Viruses** |  |
| Adenovirus 40/41 | Jothikumar, N.; Cromeans, T. L.; Hill, V. R.; Lu, X.; Sobsey, M. D.; Erdman, D. D. Quantitative Real-Time PCR Assays for Detection of Human Adenoviruses and Identification of Serotypes 40 and 41. *Appl. Environ. Microbiol.* **2005**, *71* (6), 3131–3136. |
| Astrovirus | Liu, J.; Kibiki, G.; Maro, V.; Maro, A.; Kumburu, H.; Swai, N.; Taniuchi, M.; Gratz, J.; Toney, D.; Kang, G.; et al. Multiplex Reverse Transcription PCR Luminex Assay for Detection and Quantitation of Viral Agents of Gastroenteritis. *J. Clin. Virol.* **2011**, *50* (4), 308–313. |
| Norovirus GI | Jothikumar, N.; Lowther, J. A.; Henshilwood, K.; Lees, D. N.; Hill, V. R.; Vinjé, J. Rapid and Sensitive Detection of Noroviruses by Using TaqMan-Based One-Step Reverse Transcription-PCR Assays and Application to Naturally Contaminated Shellfish Samples. *Appl. Environ. Microbiol.* **2005**, *71* (4), 1870–1875. |
| Norovirus GII | Kageyama, T.; Kojima, S.; Shinohara, M.; Uchida, K.; Fukushi, S.; Hoshino, F. B.; Takeda, N.; Katayama, K. Broadly Reactive and Highly Sensitive Assay for Norwalk-like Viruses Based on Real-Time Quantitative Reverse Transcription-PCR. *J. Clin. Microbiol.* **2003**, *41* (4), 1548–1557. |
| Rotavirus A | Jothikumar, N.; Kang, G.; Hill, V. R. Broadly Reactive TaqMan® Assay for Real-Time RT-PCR Detection of Rotavirus in Clinical and Environmental Samples. *J. Virol. Methods* **2009**, *155* (2), 126–131. |
| Sapovirus I/II/IV | Liu, J.; Gratz, J.; Amour, C.; Kibiki, G.; Becker, S.; Janaki, L.; Verweij, J. J.; Taniuchi, M.; Sobuz, S. U.; Haque, R.; et al. A Laboratory-Developed Taqman Array Card for Simultaneous Detection of 19 Enteropathogens. *J. Clin. Microbiol.* **2013**, *51* (2), 472–480. |
| Sapovirus V | Liu, J.; Gratz, J.; Amour, C.; Kibiki, G.; Becker, S.; Janaki, L.; Verweij, J. J.; Taniuchi, M.; Sobuz, S. U.; Haque, R.; et al. A Laboratory-Developed Taqman Array Card for Simultaneous Detection of 19 Enteropathogens. *J. Clin. Microbiol.* **2013**, *51* (2), 472–480. |
| **Protozoa** |  |
| *Cryptosporidium parvum* | Jothikumar, N.; da Silva, A. J.; Moura, I.; Qvarnstrom, Y.; Hill, V. R. Detection and Differentiation of Cryptosporidium Hominis and Cryptosporidium Parvum by Dual TaqMan Assays. *J. Med. Microbiol.* **2008**, *57* (9), 1099–1105. |
| *Entamoeba histolytica* | Verweij, J. J.; Blangé, R. A.; Templeton, K.; Schinkel, J.; Brienen, E. A. T.; van Rooyen, M. A. A.; van Lieshout, L.; Polderman, A. M. Simultaneous Detection of Entamoeba Histolytica, Giardia Lamblia, and Cryptosporidium Parvum in Fecal Samples by Using Multiplex Real-Time PCR. *J. Clin. Microbiol.* **2004**, *42* (3), 1220–1223. |
| *Giardia duodenalis* | Verweij, J. J.; Blangé, R. A.; Templeton, K.; Schinkel, J.; Brienen, E. A. T.; van Rooyen, M. A. A.; van Lieshout, L.; Polderman, A. M. Simultaneous Detection of Entamoeba Histolytica, Giardia Lamblia, and Cryptosporidium Parvum in Fecal Samples by Using Multiplex Real-Time PCR. *J. Clin. Microbiol.* **2004**, *42* (3), 1220–1223. |
| ***Helminth*** |  |
| *Ascaris lumbricoides* | Wiria, A. E.; Prasetyani, M. A.; Hamid, F.; Wammes, L. J.; Lell, B.; Ariawan, I.; Uh, H. W.; Wibowo, H.; Djuardi, Y.; Wahyuni, S.; et al. Does Treatment of Intestinal Helminth Infections Influence Malaria? Background and Methodology of a Longitudinal Study of Clinical, Parasitological and Immunological Parameters in Nangapanda, Flores, Indonesia (ImmunoSPIN Study). *BMC Infect. Dis.* **2010**, *10* (1), 77. |
| *Trichuris trichiuria* | Pilotte, N.; Papaiakovou, M.; Grant, J. R.; Bierwert, L. A.; Llewellyn, S.; McCarthy, J. S.; Williams, S. A. Improved PCR-Based Detection of Soil Transmitted Helminth Infections Using a Next-Generation Sequencing Approach to Assay Design. *PLoS Negl. Trop. Dis.* **2016**, *10* (3), e0004578. |
