# Supplementary material for: Impact of sampling depth on pathogen detection in pit latrines
Source: PLoS Negl Trop Dis. 2021 Mar 2;15(3):e0009176. doi: 10.1371/journal.pntd.0009176 (PMC7954291; doi:10.1371/journal.pntd.0009176)
Supplement: S2 Table — (DOCX) [file pntd.0009176.s008.docx]

Table S2. Interpretation of gene targets on the TAC

| Target | Gene Targeted | Interpretation |
| --- | --- | --- |
| **Bacteria** |  |  |
| *Campylobacter coli* | *cadF* gene | If either was detected, call as *Campylobacter coli/jejuni* positive |
| *Campylobacter jejuni* | *cadF gene* |  |
| *Clostridium difficile* (*tcdA*) | *tcdA* gene | If either was detected, call as *Clostridium difficile* positive |
| *Clostridium difficile* (*tcdB)* | *tcdB gene* |  |
| *EIEC / Shigella* (*ipaH*) | *ipaH* gene | If detected, call as *Shigella*/EIEC positive |
| EAEC (*aaiC*) | *aaiC* gene | If either was detected, call as EAEC positive |
| EAEC (*aatA*) | *aatA* gene |  |
| EPEC (*bfpA*) | *bfpA* gene | If either was detected, call as EPEC positive |
| EPEC (*eae*) | *eae* gene |  |
| ETEC-LT | *LT* gene | If either was detected, call as ETEC positive |
| ETEC-ST | *STh/STp* |  |
| *Salmonella* spp. | *invA* gene | If detected, call as *Salmonella spp.* positive |
| Shiga-like toxin 1 (stx1) | *stx_1_* gene | If either was detected, call as STEC positive |
| Shiga-like toxin 2 (stx2) | *stx_2_* gene |  |
| *Vibrio cholerae* | *toxR* gene | If detected, call as *Vibrio cholerae* positive |
| *Yersinia* spp. | *lysP* gene | If detected, call as *Yersinia spp.* positive |
| **Viruses** |  |  |
| Adenovirus 40/41 | *Fiber* gene | If detected, call as Adenovirus 40/41 positive |
| Astrovirus | *Capsid* gene | If detected, call as Astrovirus positive |
| Norovirus GI | *ORF1-ORF2* gene | If either was detected, call as Norovirus GI/GII positive |
| Norovirus GII | *ORF1-ORF2* gene |  |
| Rotavirus A | *NSP3* gene | If detected, call as Rotavirus positive |
| Sapovirus I/II/IV | *RdRp* gene | If either was detected, call as Sapovirus positive |
| Sapovirus V | *RdRp* gene |  |
| ***Protozoa*** |  |  |
| *Cryptosporidium parvum* | *18S* | If detected, call as *Cryptosporidium parvum* positive |
| *Entamoeba histolytica* | *18S* | If detected, call as *Entamoeba histolytica* positive |
| *Giardia duodenalis* | *18S* | If detected, call as *Giardia duodenalis* positive |
| ***Helminth*** |  |  |
| *Ascaris lumbricoides* | *18S* | If detected, call as *Ascaris lumbricoides* positive |
| *Trichuris trichiuria* | *ITS1* | If detected, call as *Trichuris trichiuria* positive |
