# Supplementary material for: Impact of sampling depth on pathogen detection in pit latrines
Source: PLoS Negl Trop Dis. 2021 Mar 2;15(3):e0009176. doi: 10.1371/journal.pntd.0009176 (PMC7954291; doi:10.1371/journal.pntd.0009176)
Supplement: S3 Table — (DOCX) [file pntd.0009176.s009.docx]

Table S3. Co-detection by depth

|  | Distribution of co-detections | | | |
| --- | --- | --- | --- | --- |
| Pathogen | 3/3 depths | 2/3 depths | 1/3 depths | 0/3 depths |
| **Bacteria** |  |  |  |  |
| EAEC | 91% (30/33) | 9.1% (3/33) | 0% (0/33) | 0% (0/33) |
| ETEC | 45% (15/33) | 39% (13/33) | 9.1% (3/33) | 6.1% (2/33) |
| *Shigella*/EIEC | 48% (16/33) | 18% (6/33) | 18% (6/33) | 15% (5/33) |
| EPEC | 3.0% (1/33) | 21% (7/33) | 42% (14/33) | 33% (11/33) |
| *Campylobacter coli/jejuni* | 0% (0/33) | 21% (7/33) | 33% (11/33) | 45% (15/33) |
| STEC | 0% (0/33) | 3.0% (1/33) | 18% (6/33) | 79% (26/33) |
| *C. difficile* | 0% (0/33) | 6.1% (2/33) | 12% (4/33) | 82% (27/33) |
| *Yersinia* spp. | 3.0% (1/33) | 3.0% (1/33) | 6.1% (2/33) | 88% (29/33) |
| *Salmonella* | 3.0% (1/33) | 0% (0/33) | 12% (4/33) | 85% (28/33) |
| *Vibrio cholerae* | 0% (0/33) | 0% (0/33) | 0% (0/33) | 100% (33/33) |
| **Viruses** |  |  |  |  |
| Norovirus GI/GII | 82% (27/33) | 18% (6/33) | 0% (0/33) | 82% (27/33) |
| Astrovirus | 73% (24/33) | 15% (5/33) | 9.1% (3/33) | 73% (24/33) |
| Adenovirus 40/41 | 42% (14/33) | 18% (6/33) | 27% (9/33) | 42% (14/33) |
| Sapovirus I/II/IV/V | 30% (10/33) | 39% (13/33) | 24% (8/33) | 30% (10/33) |
| Rotavirus A | 27% (9/33) | 18% (6/33) | 15% (5/33) | 39% (13/33) |
| **Protozoa** |  |  |  |  |
| *Giardia duodenalis* | 82% (27/33) | 12% (4/33) | 6.1% (2/33) | 0% (0/33) |
| *Cryptosporidium parvum* | 0% (0/33) | 12% (4/33) | 21% (7/33) | 67% (22/33) |
| *Entamoeba histolytica* | 0% (0/33) | 0% (0/33) | 0% (0/33) | 100% (33/33) |
| **Helminths** |  |  |  |  |
| *Ascaris lumbricoides* | 39% (13/33) | 33% (11/33) | 12% (4/33) | 15% (5/33) |
| *Trichuris trichiuria* | 0% (0/33) | 0% (0/33) | 0% (0/33) | 100% (33/33) |

Note: Taxa are ordered by their prevalence rank for each group
