# Supplementary material for: Impact of sampling depth on pathogen detection in pit latrines
Source: PLoS Negl Trop Dis. 2021 Mar 2;15(3):e0009176. doi: 10.1371/journal.pntd.0009176 (PMC7954291; doi:10.1371/journal.pntd.0009176)
Supplement: Attachment — Submitted filename: PNTD-D-20-02024_review.pdf [file pntd.0009176.s010.pdf]

PLOS Neglected Tropical Diseases

Impact of sampling depth on enteric pathogen detection in pit latrines

--Manuscript Draft--

**Manuscript Number:**	PNTD-D-20-02024
**Full Title:**	Impact of sampling depth on enteric pathogen detection in pit latrines
**Short Title:**	Enteric pathogens and latrine sampling depth
**Article Type:**	Research Article
**Keywords:**	enteric pathogens; sanitation; surveillance; WASH
**Abstract:**	Wastewater based epidemiology (WBE) is increasingly used to provide decision makers with actionable data about community health. WBE efforts to date have primarily focused on sewer-transported wastewater in high-income countries, but at least 1.8 billion people in low- and middle-income countries (LMIC) use onsite sanitation systems such as pit latrines and septic tanks. Like wastewater, fecal sludges from such systems also offer the potential for epidemiological modeling. To evaluate the spatial distribution of enteric pathogens inside pit latrines – which could inform sampling methods for WBE in LMICs not covered by sewers – we collected fecal sludges from the top, middle, and bottom of 33 pit latrines in urban and peri-urban Malawi and analyzed the 99 samples for 20 common enteric pathogens via multiplex quantitative reverse transcription PCR. Using logistic regression adjusted for household population, latrine sharing, the presence of a concrete floor or slab, water source, and anal cleansing materials, we found no significant difference in the odds of detecting the 20 pathogens assessed in the middle samples (adjusted odds ratio, aOR = 1.1; 95% confidence interval = 0.73, 1.6) and top samples (aOR = 0.80, 95% CI = 0.54, 1.2) compared to the bottom samples. These results suggest that, for the purposes of a WBE approach, pit latrine sampling depth does not strongly influence the odds of detecting molecular markers of enteric pathogens. A single top, or a composite of top samples, may be preferred as it represents the freshest stool contributed to the pit and is the easiest to collect.
**Additional Information:**	
**Question**	**Response**
**Financial Disclosure**<br><br>Enter a financial disclosure statement that describes the sources of funding for the work included in this submission. Review the submission guidelines for detailed requirements. View published research articles from *PLOS NTDs* for specific examples.<br><br>This statement is required for submission and **will appear in the published article** if the submission is accepted. Please make sure it is accurate.	This study was funded in part by the Bill and Melinda Gates Foundation (www.gatesfoundation.org) grant OPP1137224 to JB. The funders had no role in study design, data collection and analysis, decision to publish, or preparation of the manuscript.

**Unfunded studies**<br><br>Enter: *The author(s) received no specific funding for this work.*<br><br>**Funded studies**<br><br>Enter a statement with the following details:<br>• Initials of the authors who received each award<br>• Grant numbers awarded to each author<br>• The full name of each funder<br>• URL of each funder website<br>• Did the sponsors or funders play any role in the study design, data collection and analysis, decision to publish, or preparation of the manuscript?<br>• **NO** - Include this sentence at the end of your statement: *The funders had no role in study design, data collection and analysis, decision to publish, or preparation of the manuscript.*<br>• **YES** - Specify the role(s) played.<br><br>* typeset	
**Competing Interests**<br><br>Use the instructions below to enter a competing interest statement for this submission. On behalf of all authors, disclose any competing interests that could be perceived to bias this work—acknowledging all financial support and any other relevant financial or non-financial competing interests.<br><br>This statement **will appear in the published article** if the submission is accepted. Please make sure it is accurate. View published research articles from *PLOS NTDs* for specific examples.	The authors have declared that no competing interests exist.

Powered by Editorial Manager® and ProduXion Manager® from Aries Systems Corporation

**NO authors have competing interests**<br><br>Enter: *The authors have declared that no competing interests exist.*<br><br>**Authors with competing interests**<br><br>Enter competing interest details beginning with this statement:<br><br>*I have read the journal's policy and the authors of this manuscript have the following competing interests: [insert competing interests here]*<br><br>* typeset	
**Data Availability**<br><br>Authors are required to make all data underlying the findings described fully available, without restriction, and from the time of publication. PLOS allows rare exceptions to address legal and ethical concerns. See the PLOS Data Policy and FAQ for detailed information.<br><br>A Data Availability Statement describing where the data can be found is required at submission. Your answers to this question constitute the Data Availability Statement and **will be published in the article**, if accepted.	Yes - all data are fully available without restriction

Powered by Editorial Manager® and ProduXion Manager® from Aries Systems Corporation

**Important:** Stating 'data available on request from the author' is not sufficient. If your data are only available upon request, select 'No' for the first question and explain your exceptional situation in the text box.<br><br>Do the authors confirm that all data underlying the findings described in their manuscript are fully available without restriction?	
**Describe where the data may be found in full sentences. If you are copying our sample text, replace any instances of XXX with the appropriate details.**<br><br>• If the data are **held or will be held in a public repository**, include URLs, accession numbers or DOIs. If this information will only be available after acceptance, indicate this by ticking the box below. For example: *All XXX files are available from the XXX database (accession number(s) XXX, XXX.).*<br>• If the data are all contained **within the manuscript and/or Supporting Information files**, enter the following: *All relevant data are within the manuscript and its Supporting Information files.*<br>• If neither of these applies but you are able to provide **details of access elsewhere**, with or without limitations, please do so. For example:<br><br>*Data cannot be shared publicly because of [XXX]. Data are available from the XXX Institutional Data Access / Ethics Committee (contact via XXX) for researchers who meet the criteria for access to confidential data.*<br><br>*The data underlying the results presented in the study are available*	All data used in this paper is available at a dedicated data repository at Open Science Framework (OSF.io). We will provide the link upon acceptance.

from (include the name of the third party and contact information or URL).<br>• This text is appropriate if the data are owned by a third party and authors do not have permission to share the data.<br><br>* typeset	
Additional data availability information:	Tick here if the URLs/accession numbers/DOIs will be available only after acceptance of the manuscript for publication so that we can ensure their inclusion before publication.

Powered by Editorial Manager® and ProduXion Manager® from Aries Systems Corporation

Manuscript
Click here to access/download;Manuscript;Malawi Fecal Sludge_PLOS NTDs.docx

Full Title: Impact of sampling depth on enteric pathogen detection in pit latrines

Short Title: Enteric pathogens and latrine sampling depth

Capone, Drew[1,2]; Chigwechokha, Petros[3]; de los Reyes III, Francis L.[4]; Holm, Rochelle H. [5,6]; Risk, Benjamin B.[7]; Tilley, Elizabeth[8,9]; Brown, Joe[2]

1. Civil and Environmental Engineering, Georgia Institute of Technology, 311 Ferst Drive, Atlanta, Georgia, United States of America
2. Department of Environmental Sciences and Engineering, Gillings School of Public Health, University of North Carolina at Chapel Hill, 135 Dauer Dr., Chapel Hill, North Carolina, United States
3. Directorate of Research, Malawi University of Science and Technology, Malawi
4. Department of Civil, Construction, and Environmental Engineering, North Carolina State University, Raleigh, North Carolina, United States
5. Centre of Excellence in Water and Sanitation, Mzuzu University, P/Bag 201, Mzuzu 2, Malawi
6. Christina Lee Brown Environmental Institute, University of Louisville, 302 E. Muhammad Ali Blvd., Louisville, KY 40202, United States
7. Department of Biostatistics and Bioinformatics, Emory University, Atlanta, Georgia, United States of America
8. Department of Environmental Health, University of Malawi, The Polytechnic, Blantyre, Malawi
9. Department of Sanitation, Water and Solid Waste for Development, Swiss Federal Institute of Aquatic Science and Technology, Duebendorf, Switzerland

Word Count: 3166 words + 3 tables (no word limit)

Supplemental Information: 4 figures, 2 texts, 3 tables

Abstract

Wastewater based epidemiology (WBE) is increasingly used to provide decision makers with actionable data about community health. WBE efforts to date have primarily focused on sewer-transported wastewater in high-income countries, but at least 1.8 billion people in low- and middle-income countries (LMIC) use onsite sanitation systems such as pit latrines and septic tanks. Like wastewater, fecal sludges from such systems also offer the potential for epidemiological modeling. To evaluate the spatial distribution of enteric pathogens inside pit latrines – which could inform sampling methods for WBE in LMICs not covered by sewers – we collected fecal sludges from the top, middle, and bottom of 33 pit latrines in urban and peri-urban Malawi and analyzed the 99 samples for 20 common enteric pathogens via multiplex quantitative reverse transcription PCR. Using logistic regression adjusted for household population, latrine sharing, the presence of a concrete floor or slab, water source, and anal cleansing materials, we found no significant difference in the odds of detecting the 20 pathogens assessed in the middle samples (adjusted odds ratio, aOR = 1.1; 95% confidence interval = 0.73, 1.6) and top samples (aOR = 0.80, 95% CI = 0.54, 1.2) compared to the bottom samples. These results suggest that, for the purposes of a WBE approach, pit latrine sampling depth does not strongly influence the odds of detecting molecular markers of enteric pathogens. A single top, or a composite of top samples, may be preferred as it represents the freshest stool contributed to the pit and is the easiest to collect.

Author Summary

Almost two billion people defecate into onsite sanitation technologies such as pit latrines. Evidence suggests these individuals are often at a disproportionately high risk of enteric infection compared to people served by piped sewerage. Public health surveillance in these high-risk communities could test for pathogens in fecal sludges – the combined feces from the individual contributing waste to latrines – to inform which pathogens are circulating in the community. Such data could inform vaccination campaigns and interventions to limit the transmission of these pathogens, including eradication efforts. A better understanding of how enteric pathogen detection varies across different pit latrine depths would be useful to inform methods for fecal sludge collection. To explore this gap, we collected fecal sludges from the top, middle, and bottom of pit latrines in Malawi. We found pit latrine depth did not significantly affect enteric pathogen detection. However, the top samples may be preferred due to their ease of collection and because the most recently deposited stools are likely of greatest interest in surveillance efforts.

Introduction

Wastewater-based epidemiology (WBE) is a rapid and scalable approach with the potential to conduct community health surveillance in near real-time (1–4). As a complement to on-going clinical testing, WBE has provided actionable data to decision makers in global polio eradication efforts (5–8) and in response to the COVID-19 pandemic (2,9–12). Other WBE studies, which have primarily focused on wastewater from high-income countries, investigated illicit and licit drug use (13,14), antibiotic resistance (15), the community microbiome (16), and used biomarkers to predict population socioeconomic characteristics (17,18).

In low- and middle-income countries (LMIC), onsite sanitation technologies – including pit latrines and septic tanks – serve at least 1.8 billion people (19,20). In most cities across the Global South, sewers serve a small fraction of the population and often do not extend to unplanned urban settlements where the burden of enteric disease is disproportionately high (21,22). To monitor common enteric pathogens circulating among these populations of interest, sampling fecal sludges from onsite sanitation technologies is a plausible and logical extension of wastewater-based epidemiology (23). Fecal sludges offer a pooled, noninvasive, and generally anonymized, community sampling approach that is logistically far easier than collection of biological specimens from human participants. Indeed, a more inclusive term for these approaches may be “waste-based epidemiology”, which retains the WBE acronym.

Similar to wastewater, a sample of fecal sludge represents the pooled waste from a group of individual users, but represents fewer people (24,25). Onsite sanitation technologies, however, are used by one or several households and provide data at a finite geospatial resolution, which in some instances may be preferred over a snapshot of an entire sewershed (i.e., the area served by a given sewer network). Wastewater is often sampled from a single point in the sewershed to

conduct WBE (15), but collection of fecal sludges from multiple pit latrines or septic tanks is likely necessary (23). There is also the potential to collect fecal sludge samples from other points in the sludge management chain, including from sites receiving waste from many latrines. Such approaches may be feasible in cities that remain largely unserved by sewerage (26). Because users of onsite sanitation may be higher risk for enteric infections compared with those served by sewerage (27,28) surveillance of fecal sludges from such systems is critical to obtain data on those most at risk.

Ideally, a representative sample of fecal sludge from an onsite system would be a composite of the feces from all individuals contributing waste to the system and free of solid waste. This is challenging, however, because sludges in pit latrines are often not well mixed and often contain refuse (29). The extent of mixing that occurs is not well characterized, but likely depends on multiple factors including latrine type, operation, emptying frequency, and the level of the water table (25). For example, pour-flush latrines, or latrines that receive greywater as an input, may experience greater mixing compared to dry pit latrines which function without water.

Extending the principles of WBE to onsite sanitation technologies requires standardized methods for sampling. The physical (30,31) and microbial (31–33) dynamics of fecal sludges have increasingly been studied, but attempts to characterize the spatial distribution of enteric pathogens in onsite technologies have been primarily limited to fecal indicator bacteria (34). It is unclear how the intra-pit depth (e.g. top, middle, bottom of the pit) of fecal sludge sampling may impact the detection of enteric pathogens in high burden settings. To explore this gap, the aim of this study was to compare the presence and type of enteric pathogens between the top, middle, and bottom of pit latrines in urban and peri-urban Malawi. Understanding how enteric pathogen detection varies with pit depth may inform sampling methods for pathogen surveillance in

LMICs, which could provide actionable data to guide prioritization of public health interventions such as vaccination (35), mass drug administration (36), or improved water, sanitation, and hygiene (WASH) (37).

Methods

We purposively enrolled participants in urban Blantyre and peri-urban Mzuzu, Malawi based on the condition of their onsite sanitation technology. The following criteria were used for enrollment: (1) the presence of a pit latrine with a concrete or wood plank floor or slab, (2) a lined pit below ground, (3) drop hole large enough for a Gulper (a type of manual pump used to empty pit latrines)(25) to empty, (4) sufficiently watery sludge capable of being pumped by a Gulper, and (5) less than 1 meter of space between the top of the fecal sludge and the latrine floor.

Sample Collection

We collected fecal sludge from the top, middle, and bottom of each pit in the urban Ndirande neighborhood in Blantyre and the peri-urban areas surrounding Mzuzu, Malawi from November to December 2018, which is the beginning of the rainy season. First, a sludge sample was scooped from the top of each pit using a disposable hand scoop placed through the squat hole (Figure S1). If there was visible solid waste in the pit that was likely to clog the Gulper, the waste was carefully removed using a metal pitchfork while attempting to minimize any mixing of the pit contents (Figure S2). Next, a Gulper was lowered into the pit until the full length of the Gulper (approximately 1.5m) was submerged (Figure S3). The first “pump” of sludge was assumed to have been the sludge that filled the Gulper as it was lowered and was not saved. Then, the second “pump” of sludge was collected in a bucket and considered as the sludge from the bottom of the pit. When approximately half of the pit was emptied, another “pump” of sludge

was collected into a separate bucket and considered as the sludge to be from the middle of the pit. Finally, the sample from each depth was aliquoted into three sterile 15mL centrifuge tubes which were transported to the lab at Mzuzu University (Mzuzu samples) or the University of Malawi (Blantyre samples) (Figure S4).

Data Collection

On the same day as sample collection from the household's latrine, we interviewed an adult member of the household regarding the characteristics and typical use of their pit latrine. Direct observation of the characteristics and condition of the latrine was guided by a checklist.

Sample Processing

We stored samples temporarily at 4°C until all samples had been collected (about ten days). Then, the 15mL tubes were centrifuged at 2000 RPM for 5 minutes, the supernatant was poured off, 0.5g of each pellet was transferred into a sterile 2mL centrifuge tube, and then 0.75mL of UNEX buffer (Microbiologics, St. Cloud, Minnesota, USA) was added to each tube. Samples were shipped at ambient conditions to North Carolina State University at Raleigh (Raleigh, North Carolina) and then transported on ice to Georgia Institute of Technology (Atlanta, Georgia), where they were transferred to a -80° C freezer for storage until extraction.

We extracted total nucleic acids from 100 mg of the fecal sludge-UNEX buffer mixture using the Qiagen 96 Virus QIAcube HT Kit (Qiagen, Hilden, Germany) (Text S1) (23,38). Samples were spiked with MS2 as an extraction control and one negative extraction control was included per day of extractions. Then, nucleic acids were analyzed with a custom TaqMan Array Card (TAC) – a multiplex quantitative reverse transcription PCR platform – that tested for 20 enteric pathogens in duplicate (23) (Text S2, Table S1, Table S2). Each TAC included a positive and negative control; positive controls were gene targets inserted into plasmids (39). Exponential curves and multicomponent plots were examined for each target – including comparison with the

positive control – to validate positive amplification. Any sample exhibiting positive amplification in either duplicate well before a Cq of 40 was called positive (23).

Data analysis

We compared individual pathogen detection between depths using the Jaccard similarity coefficient (40) (i.e., the intersection of detections over the union). Because it excludes instances where a pathogen is not detected in either depth, this coefficient does not bias the estimate upwards for infrequently detected pathogens. For example, from the matched 33 top and 33 middle depth samples, if we were to detect an individual pathogen at both depths 20 times, only in the top 4 times, only in the middle 6 times, and in neither sample 3 times, the Jaccard similarity coefficient is 67% (Equation 1). Further, we calculated 95% confidence intervals around the Jaccard similarity coefficient by bootstrapping our data with 10,000 iterations.

(1) $$J_X = 20 \div (20 + 4 + 6) = 67\%$$

We used logistic regression to compare overall pathogen detection between pit latrine depths which generated unadjusted and adjusted prevalence odds ratios (OR, aOR). The binary presence or absence of each of the 20 pathogens assessed was our response variable, such that each observation represented the presence or absence of a particular pathogen at a given depth and latrine; depth as a categorical variable (bottom, middle, top) was our exposure variable. We *a priori* selected potential confounders from our household survey questionnaire based on the potential to impact pathogen detection in fecal sludges, estimating both unadjusted and adjusted effects. Selected confounders included household population, whether the pit latrine was shared with other households, the presence of a concrete floor or slab, a categorical variable for water source (public tap, tap inside the compound, tap inside the house), and the most frequent anal cleansing material used (water or paper). We included random effects for pit latrine (33 levels),

pathogen (20 levels), and their interaction. To fit regression models we used generalized linear mixed-effects models (GLMM) with a binomial distribution (logit link) (41). We analyzed data in R version 4.0.0 (R Foundation for Statistical Computing, Vienna, Austria).

Ethics

Participation was based on informed, written consent by a household member over the age 18. This study received ethical approval and a material transfer agreement from the Republic of Malawi National Health Sciences Research Committee (Protocol 17/09/1915).

Results

We collected fecal sludge from the top, middle, and bottom of 3 dry pit latrines in peri-urban Blantyre and from 30 dry pit latrines in peri-urban Mzuzu, Malawi for a total of 99 samples. Most of the latrines had a concrete floor or portable slab (85%, [28/33]) and the rest did not have a slab (15%, [5/33]). The mean number of people per household was 7.6 (standard deviation = 3.4, range = 3-18) and 74% (24/33) of households reported sharing the latrine with other households.

We commonly detected bacteria, viruses, and protozoa at each of the three pit latrine depths (Table 1), though we did not detect *Vibrio cholerae* or *Entamoeba histolytica*. Among the helminths, we did not detect *Trichuris* from any sample, but detected *Ascaris* in most samples (Table 1, Table 2).

Table 1. Detection of pathogens in fecal sludges (n = 33 pits)

	Median number of pathogens detected (IQR)			Prevalence (95% CI)		
	**Top**	**Middle**	**Bottom**	**Top**	**Middle**	**Bottom**
**Bacteria (out of 10)**	3 (1)	3 (2)	3 (2)	100%	100%	100%
**Viruses (out of 5)**	3 (2)	4 (1)	4 (2)	100%	100%	100%
**Protozoa (out of 3)**	1 (0)	1 (0)	1 (0)	88% (77%, 99%)	94% (86%, 100%)	94% (86%, 100%)
**Helminths (out of 2)**	1 (1)	1 (0)	1 (1)	55% (37%, 72%)	76% (61%, 91%)	67% (50%, 83%)

**All pathogens (out of 20)**	8 (2)	9 (4)	8 (3)	100%	100%	100%

Note: Pathogens detected via RT-qPCR using a Custom TaqMan array card. IQR: interquartile range

Table 2. Pathogen and indicator detection by pit latrine depth.

	Pathogen detection			Jaccard similarity coefficient*		
Pathogens	Bottom (95% CI)	Middle (95% CI)	Top (95% CI)	Top-Middle	Middle-Bottom	Top-Bottom
**Bacteria**						
EAEC	91% (81%, 100%)	100%	100%	97% (91%, 100%)	94% (85%, 100%)	91% (79%, 100%)
ETEC	70% (54%, 86%)	76% (61%, 91%)	79% (65%, 93%)	82% (68%, 96%)	78% (62%, 93%)	75% (58%, 90%)
Shigella/EIEC	70% (54%, 86%)	64% (47%, 80%)	67% (50%, 83%)	65% (48%, 85%)	76% (58%, 92%)	67% (47%, 83%)
EPEC	27% (12%, 43%)	33% (17%, 50%)	33% (17%, 50%)	29% (5.9%, 50%)	67% (38%, 92%)	25% (8.3%, 53%)
Campylobacter coli/jejuni	18% (4.8%, 32%)	27% (12%, 43%)	27% (12%, 43%)	29% (9.1%, 67%)	50% (18%, 82%)	36% (7.1%, 55%)
STEC	12% (0.81%, 23%)	6.1% (0%, 14%)	9.1% (0%, 19%)	25% (0%, 67%)	25% (0%, 100%)	20% (0%, 100%)
C. difficile	9.1% (0%, 19%)	6.1% (0%, 14%)	6.1% (0%, 14%)	100%	67% (0%, 100%)	67% (0%, 100%)
Yersinia spp.	6.1% (0%, 14%)	9.1% (0%, 19%)	6.1% (0%, 14%)	25% (0%, 100%)	67% (0%, 100%)	33% (0%, 100%)
Salmonella	3.0% (0%, 9.0%)	9.1% (0%, 19%)	9.1% (0%, 19%)	20% (0%, 100%)	33% (0%, 100%)	33% (0%, 67%)
Vibrio cholerae	0%	0%	0%			
**Viruses**						
Norovirus GI/GII	94% (86%, 100%)	97% (91%, 100%)	91% (81%, 100%)	88% (73%, 97%)	97% (90%, 100%)	85% (76%, 97%)
Astrovirus	88% (77%, 99%)	88% (77%, 99%)	82% (68%, 95%)	81% (59%, 90%)	93% (83%, 100%)	75% (66%, 94%)
Sapovirus I/II/IV/V	67% (50%, 83%)	67% (50%, 83%)	58% (40%, 75%)	64% (50%, 86%)	67% (48%, 84%)	68% (44%, 83%)
Adenovirus 40/41	67% (50%, 83%)	61% (44%, 78%)	64% (47%, 80%)	52% (46%, 83%)	68% (48%, 86%)	65% (33%, 71%)
Rotavirus A	48% (31%, 65%)	48% (31%, 65%)	36% (20%, 53%)	55% (32%, 79%)	79% (57%, 91%)	63% (25%, 70%)
**Protozoa**						
Giardia duodenalis	94% (86%, 100%)	94% (86%, 100%)	88% (77%, 99%)	88% (70%, 94%)	94% (84%, 100%)	82% (75%, 97%)
Cryptosporidium parvum	24% (9.4%, 39%)	9.1% (0%, 19%)	12% (0.81%, 23%)	17% (0%, 50%)	10% (0%, 33%)	20% (0%, 50%)
Entamoeba histolytica	0%	0%	0%	ND	ND	ND
**STHs**						
Ascaris lumbricoides	67% (50%, 83%)	76% (61%, 91%)	55% (37%, 72%)	59% (35%, 73%)	74% (57%, 89%)	54% (41%, 78%)
Trichuris trichiuria	0%	0%	0%	ND	ND	ND
Overall				67% (62%, 72%)	78% (73%, 82%)	67% (61%, 72%)

Note: ND: non-detect. *C. difficile*: *Clostridium difficile*. EAEC: *Enteroaggregative E. coli*. EIEC: *Enteroinvasive E. coli*. ETEC: *Enterotoxigenic E. coli*. EPEC: *Enteropathogenic E. coli*. STEC: shiga-toxin producing *E. coli*.

*Size of the intersection of matched detections divided by the size of the union of detections. For example, if we detected *Giardia* in both the top and the middle fecal sludge samples 28 times, only in the top 1 time, only in the middle 3 times, and did not detect *Giardia* in either sample 1 time: $J_{Giardia}$ = (28) / (28 + 1 + 3) = 88%. 95% confidence intervals calculated by bootstrapping with 10,000 iteration

Inter-latrine results

At each depth, we observe the same rank order prevalence for the five most common bacterial pathogens: *Enteroaggregative E. coli* (EAEC), *Enterotoxigenic E. coli* (ETEC), *Shigella*/*Enteroinvasive E. coli* (EIEC), *Enteropathogenic E. coli* (EPEC), and *Campylobacter coli*/*jejuni*. Likewise, we detected all five viral pathogens in the same rank order at all three depths, except for sapovirus and adenovirus 40/41 in the top samples, and we observed the same rank order detection among all three protozoan pathogens and both helminths (Table 2).

Intra-latrine results

Comparing the three sampling depths, the Jaccard similarity coefficient indicated that the bottom and middle samples were most similar ($J_{bottom,middle} = 78\%,;\ 95\%\ CI = 73\%, 82\%$), followed by the top and middle samples ($J_{top,middle} = 67\%;\ 95\%\ CI = 62\%, 72\%$), and top and bottom samples ($J_{top,bottom} = 67\%;\ 95\%\ CI = 61\%, 72\%$). Co-detection of a pathogen at the three depths from the same pit latrine tended to increase with increased prevalence in sludges (Table S3).

Adjusted for household population, latrine sharing, the presence of a concrete floor or slab, water source, and anal cleansing materials, there was no significant effect of depth on the prevalence of a pathogen on TAC (X^2(df = 2) = 2.4, p = 0.30). The odds of detecting the 20 pathogens on TAC at the middle depth compared to the bottom was nearly one (aOR = 1.1; 95% confidence interval = 0.73, 1.6) and also nearly one for the top compared to the bottom (aOR = 0.80, 95% CI = 0.54, 1.2) (Table 3). This result indicates that we did not observe a significant difference in detected pathogens between the three depths. Further, a 1-person increase in household population was associated with increased odds of pathogen detection (aOR = 1.2, 95% CI = 1.1, 1.3); latrines shared among multiple households were not associated with pathogen

detection compared to private latrines (aOR = 1.3, 95% CI: 0.68, 2.3); both having a water source inside the compound (aOR = 0.46, 95% CI: 0.23, 0.93) and inside the home (aOR = 0.13 , 95% CI = 0.05, 0.36) were associated with reduced odds of pathogen detection compared to households who reported a public tap as their water source; using water for anal cleansing was not associated with pathogen detection compared to using toilet paper (aOR = 1.8, 95% CI: 0.79, 4.2); and latrines with concrete floors or slabs were not associated with pathogen detection (aOR = 0.89, 95% CI: 0.42, 1.9) compared to latrines without concrete floors or slabs (Table 3).

Table 3. Odds of detecting the 20 pathogens on TAC in samples from different pit depths

**Variable**	**Reference**	**OR**	**aOR**
Depth: middle	Bottom	1.1 (0.73, 1.6)	1.1 (0.73, 1.6)
Depth: top		0.81 (0.54, 1.2)	0.80 (0.54, 1.2)
Household population	1-person increase	**1.2 (1.1, 1.3)**	**1.2 (1.1, 1.3)**
Shared status: shared by multiple households	Private latrine	1.8 (0.86, 3.9)	1.3 (0.68, 2.3)
Water source: inside the compound	Communal public tap	0.79 (0.36, 1.7)	**0.46 (0.23, 0.93)**
Water source: inside the house		**0.28 (0.10, 0.76)**	**0.13 (0.05, 0.36)**
Anal cleansing: Water	Toilet paper	0.76 (0.29, 2.0)	1.8 (0.79, 4.2)
Concrete floor or slab present	No slab	0.63 (0.24, 1.7)	0.89 (0.42, 1.9)

Note: OR: odds ratio. aOR: adjusted odds ratio. Bold indicates $p \leq 0.05$

Discussion

We observed a high degree of homogeneity in enteric pathogen detection between fecal sludge samples collected at the top, middle, and bottom of pit latrines in urban and peri-urban Malawi. Rank order prevalence, regression analysis and the Jaccard similarity coefficient indicated the pathogens detected in the bottom of latrines were not substantially different compared to pathogens detected in the middle and top of the same pits. The three metrics suggested pathogens detected in the middle and bottom samples were more closely aligned with each other than with the top samples. This finding suggests that a single fecal sludge sample taken from a standardized depth may be representative of the enteric pathogens present in the entire pit. However, to conduct near real-time pathogen surveillance the choice of depth remains important when deciding between fresher sludge collected at the top of the pit or older sludge collected at the middle or bottom of the pit, and practically speaking the ease of *in-situ* sample collection.

Though we lacked matched stools for comparison, the pathogens we identified are known endemic enteric pathogens from Malawi based on etiological studies of diarrheal diseases, and their presence in fecal sludges was expected (42–45). For example, we frequently detected EAEC (44), Norovirus (42,43,45), *Giardia* (42,45), and *Ascaris* (46) in sludges and previous studies in Malawi also commonly detected these pathogens in children's stools. In addition, the individual pathogens we did not identify in fecal sludges aligned well with a study of 527 asymptomatic children in Blantyre, Malawi from 2013 to 2016.(44) We did not detect *Vibrio cholerae*, *Trichuris*, or *Entamoeba hystolytica* in sludges, which Iturriza-Gómara *et al*. 2019 detected in 0%, 0.19% and 1.5% of children's stools, respectively. Given our relatively small sample size, detection of these three pathogens in fecal sludges was not expected.

The frequent co-detection of pathogens in samples from the same pit may be a result of several plausible factors. First, mechanistic forces (e.g. gravity, the addition of greywater, or insect movements) may result in the homogenization of fecal sludges. We most frequently co-detected pathogens from the bottom and middle samples, which would have been subject to gravity-induced mixing for a longer period than sludges at the top. Second, as is common in other low-income settlements (22), these results indicate a high burden of enteric infection in urban and peri-urban Malawi. This could take the form of either repeated (43,47,48) or persistent infections (49,50), which may result in a relatively consistent input of pathogens over time. Finally, pumping sludge through the Gulper likely helped to homogenize the middle and bottom samples (25) and centrifuging sludges may have further helped to concentrate pathogens. These sampling and processing methods may have contributed to the frequent co-detection of pathogens.

We observed that increasing household population was associated with an increased odds of pathogen detection. This finding was expected as the more people contribute waste to a pit latrine the greater the probability that at least one person from the household would shed a specific pathogen into the pit. In contrast, a study in low-income urban Maputo, Mozambique did not find an association between pathogen detection and an increasing population within housing compound (23). In addition, we found that having a water source inside the compound or home reduced the odds of pathogen detection compared to relying on water from a public tap. Having a water source inside the home may result in increased water use for personal hygiene or consumption, which a 2015 systematic review found was generally associated with reduced gastrointestinal infection and diarrheal disease (51).

There are several important limitations to this study. First, by removing the solid waste from some pits we may have inadvertently mixed the fecal sludge and we did not sterilize the Gulper between collection of the bottom and middle samples. Mixing before sample collection and cross contamination during sampling may have occurred and might explain why we observed that these two samples were more closely aligned with each other than with the top samples. However, solid waste removal was necessary to use the Gulper, sterilization of the Gulper was not logistically practical, and the mass of fecal sludge flowing through the Gulper was substantially greater than the mass of fecal sludge that may have stuck to the walls of the Gulper tube. Next, we used a quantal assay that determined the presence or absence of each pathogen. There may be important differences in pathogen concentrations between the depths assessed, which could influence sampling methods for waste-based epidemiology efforts from pit latrines. Although we frequently co-detected pathogens, we cannot definitively explain the causal mechanism behind our findings. A next step would be to conduct tracer studies in latrines and septic tanks in LMICs to assess mixing inside these systems. Our sample processing methods used centrifugation and discarded the resulting supernatant. While we frequently detected all viral pathogens, it is plausible that these methods underestimated viral pathogen prevalence by not analyzing the supernatant as well as the solids. Further, numerous studies have characterized pathogen decay in wastewater, including the decay of nucleic acids which are detected via PCR-based methods like those used in this study. Similar decay experiments are needed for fecal sludges. As well, we did not include household wealth or income as a confounder in our regression analysis because such an assessment would have added to the length and complexity of the questionnaire. It is likely some of the significant associations we found, such as the association between pathogen detection and water source, were confounded by socioeconomic

status. Finally, as a cross sectional study, we were unable to assess pathogen signals over time, which are necessary for interpretation and use in surveillance efforts.

Though we most frequently co-detected pathogens in the matched samples from the middle and bottom of pits, sludge samples from the top of pit latrines may be desired for surveillance efforts. Operating a Gulper is messy and requires specialized training. Other sampling methods exist to collect sludge from the middle or bottom of pit latrines (23,33), but still require specialized equipment (e.g. a Wheaton sub-surface sampler). The simplicity of using a hand scoop, or scoop like device such as a Sludge Nabber (23), to collect sludges from the top of pit latrines offers an inexpensive and scalable WBE collection method. In fact, the sludge at the top of pits represents the most recent waste contribution to the pit, which is likely desired to conduct near real-time surveillance in LMICs. These techniques, combined with analysis using the TAC platform (52–54), which permits customization to detect a wide range of pathogens based on the local context (55), offers the opportunity to gather novel and actionable public health data in LMICs where onsite sanitation predominates.

Financial Support

This study was funded by the Bill and Melinda Gates Foundation (www.gatesfoundation.org) grant OPP1137224. The funders had no role in study design, data collection and analysis, decision to publish, or preparation of the manuscript.

Disclosures

The authors declare that they have no known competing financial interests or personal relationships that could have appeared to influence the work reported in this paper.

Supporting Information Captions

1. Figure S1. Hand scoop for fecal sludge collection
2. Figure S2. Solid waste removal from a pit latrine
3. Figure S3. Operating the Gulper
4. Figure S4. Aliquoting a fecal sludge sample
5. Text S1. Methodology for total nucleic acid extraction from fecal sludges.
6. Text S2. Custom TaqMan Array Card (TAC)
7. Table S1. Assays used on the custom TAC.
8. Table S2. Interpretation of gene targets on the TAC
9. Table S3. Co-detection by depth

Author List

Capone, Drew[1,2] (dcapone@ad.unc.edu); Chigwechokha, Petros[3] (pchigwe@gmail.com) ; de los Reyes, Francis L.[4] (fldelosr@ncsu.edu); Holm, Rochelle[5,6] (rochelle@rochelleholm.com); Risk, Benjamin B.[7] (benjamin.risk@emory.edu); Tilley, Elizabeth[8,9] (elizabeth.tilley@eawag.ch); Brown, Joe[1,2] (joebrown@unc.edu)

1. Civil and Environmental Engineering, Georgia Institute of Technology, 311 Ferst Drive, Atlanta, Georgia, United States of America
2. Department of Environmental Sciences and Engineering, Gillings School of Public Health, University of North Carolina at Chapel Hill, 135 Dauer Dr., Chapel Hill, North Carolina, United States
3. Directorate of Research, Malawi University of Science and Technology, Malawi
4. Department of Civil, Construction, and Environmental Engineering, North Carolina State University, Raleigh, North Carolina, United States
5. Centre of Excellence in Water and Sanitation, Mzuzu University, P/Bag 201, Mzuzu 2, Malawi
6. Christina Lee Brown Envirome Institute, University of Louisville, 302 E. Muhammad Ali Blvd., Louisville, Kentucky, United States
7. Department of Biostatistics and Bioinformatics, Emory University, Atlanta, Georgia, United States of America, 1518 Clifton Rd NE, Atlanta, Georgia 30322, United States
8. Department of Environmental Health, University of Malawi, The Polytechnic, Blantyre, Malawi
9. Department of Sanitation, Water and Solid Waste for Development, Swiss Federal Institute of Aquatic Science and Technology, Duebendorf, Switzerland

References

1. Zuccato E, Chiabrando C, Castiglioni S, Bagnati R, Fanelli R. Estimating Community Drug Abuse by Wastewater Analysis. Environ Health Perspect [Internet]. 2008 Aug;116(8):1027–32. Available from: https://ehp.niehs.nih.gov/doi/10.1289/ehp.11022

2. Bivins A, North D, Ahmad A, Ahmed W, Alm E, Been F, et al. Wastewater-Based Epidemiology: Global Collaborative to Maximize Contributions in the Fight Against COVID-19. Environ Sci Technol [Internet]. 2020 Jul 7;54(13):7754–7. Available from: https://pubs.acs.org/doi/10.1021/acs.est.0c02388

3. Sims N, Kasprzyk-Hordern B. Future perspectives of wastewater-based epidemiology: Monitoring infectious disease spread and resistance to the community level. Vol. 139, Environment International. Elsevier Ltd; 2020. p. 105689.

4. Bisseux M, Didier D, Audrey M, Christine A, Hélène PL, Jean-Luc B, et al. Monitoring of enterovirus diversity in wastewater by ultra-deep sequencing: An effective complementary tool for clinical enterovirus surveillance. Water Res. 2020 Feb 1;169:115246.

5. Anis E, Kopel E, Singer S, Kaliner E, Moerman L, Moran-Gilad J, et al. Insidious reintroduction of wild poliovirus into Israel, 2013. Eurosurveillance [Internet]. 2013 Sep 19 [cited 2019 Apr 23];18(38):20586. Available from: http://www.eurosurveillance.org/ViewArticle.aspx?ArticleId=20586

6. Hovi T, Shulman LM, Van Der Avoort H, Deshpande J, Roivainen M, De Gourville EM. Role of environmental poliovirus surveillance in global polio eradication and beyond. Epidemiol Infect. 2012;140(1):1–13.

7. Lickness JS, Gardner T, Diop OM, Chavan S, Jorba J, Ahmed J, et al. Surveillance to Track Progress Toward Polio Eradication — Worldwide, 2018–2019. MMWR Morb Mortal Wkly Rep [Internet]. 2020 May 22;69(20):623–9. Available from: http://www.cdc.gov/mmwr/volumes/69/wr/mm6920a3.htm?s_cid=mm6920a3_w

8. O'Reilly KM, Allen DJ, Fine P, Asghar H. The challenges of informative wastewater sampling for SARS-CoV-2 must be met: lessons from polio eradication. The Lancet Microbe [Internet]. 2020 Sep 1 [cited 2020 Oct 19];1(5):e189–90. Available from: https://doi.org/10.1101/2020.03.29.20045880

9. Medema G, Heijnen L, Elsinga G, Italiaander R, Brouwer A. Presence of SARS-Coronavirus-2 RNA in Sewage and Correlation with Reported COVID-19 Prevalence in the Early Stage of the Epidemic in The Netherlands. Environ Sci Technol Lett [Internet]. 2020 May 27 [cited 2020 Jun 29]; Available from: https://pubs.acs.org/doi/full/10.1021/acs.estlett.0c00357

10. Silverman AI, Boehm AB. Systematic Review and Meta-Analysis of the Persistence and Disinfection of Human Coronaviruses and Their Viral Surrogates in Water and Wastewater. Environ Sci Technol Lett [Internet]. 2020 Jun 9;acs.estlett.0c00313. Available from: https://pubs.acs.org/doi/10.1021/acs.estlett.0c00313

11. Ahmed W, Angel N, Edson J, Bibby K, Bivins A, O'Brien JW, et al. First confirmed detection of SARS-CoV-2 in untreated wastewater in Australia: A proof of concept for the wastewater surveillance of COVID-19 in the community. Sci Total Environ [Internet]. 2020 Apr 18 [cited 2020 Apr 23];138764. Available from: https://linkinghub.elsevier.com/retrieve/pii/S0048969720322816

12. Street R, Malema S, Mahlangeni N, Mathee A. Wastewater surveillance for Covid-19: An African

perspective. Sci Total Environ [Internet]. 2020 Nov;743:140719. Available from: https://linkinghub.elsevier.com/retrieve/pii/S0048969720342418

13. Thomas K V., Bijlsma L, Castiglioni S, Covaci A, Emke E, Grabic R, et al. Comparing illicit drug use in 19 European cities through sewage analysis. Sci Total Environ [Internet]. 2012 Aug;432:432–9. Available from: https://linkinghub.elsevier.com/retrieve/pii/S0048969712008959

14. Ort C, Eppler JM, Scheidegger A, Rieckermann J, Kinzig M, Sörgel F. Challenges of surveying wastewater drug loads of small populations and generalizable aspects on optimizing monitoring design. Addiction [Internet]. 2014 Mar [cited 2020 Oct 20];109(3):472–81. Available from: https://pubmed.ncbi.nlm.nih.gov/24325468/

15. Hendriksen RS, Munk P, Njage P, van Bunnik B, McNally L, Lukjancenko O, et al. Global monitoring of antimicrobial resistance based on metagenomics analyses of urban sewage. Nat Commun. 2019 Dec 1;10(1).

16. Newton RJ, McLellan SL, Dila DK, Vineis JH, Morrison HG, Murat Eren A, et al. Sewage reflects the microbiomes of human populations. MBio. 2015;6(2):1–9.

17. Choi PM, O’Brien JW, Tscharke BJ, Mueller JF, Thomas K V., Samanipour S. Population Socioeconomics Predicted Using Wastewater. Environ Sci Technol Lett [Internet]. 2020 Aug 11 [cited 2020 Sep 15];7(8):567–72. Available from: https://dx.doi.org/10.1021/acs.estlett.0c00392

18. Ort C, Lawrence MG, Reungoat J, Mueller JF. Sampling for PPCPs in wastewater systems: Comparison of different sampling modes and optimization strategies. Environ Sci Technol [Internet]. 2010 Aug 15 [cited 2020 Oct 20];44(16):6289–96. Available from: https://pubs.acs.org/doi/abs/10.1021/es100778d

19. Berendes DM, Sumner TA, Brown JM. Safely Managed Sanitation for All Means Fecal Sludge Management for at Least 1.8 Billion People in Low and Middle Income Countries. Environ Sci Technol. 2017;51(5):3074–83.

20. Graham JP, Polizzotto ML. Pit Latrines and Their Impacts on Groundwater Quality: A Systematic Review. Environmnetal Heal Perspect. 2013;5(121):521–30.

21. Peal A, Evans B, Blackett I, Hawkins P, Heymans C. Fecal sludge management: a comparative analysis of 12 cities. J Water, Sanit Hyg Dev [Internet]. 2014;4(4):563. Available from: http://washdev.iwaponline.com/cgi/doi/10.2166/washdev.2014.026

22. Knee J, Sumner T, Adriano Z, Berendes D, de Bruijn E, Schmidt W-P, et al. Risk factors for childhood enteric infection in urban Maputo, Mozambique: A cross-sectional study. Mejia R, editor. PLoS Negl Trop Dis [Internet]. 2018 Nov 12;12(11):e0006956. Available from: http://www.ncbi.nlm.nih.gov/pubmed/30419034

23. Capone D, Berendes D, Cumming O, Knee J, Nalá R, Risk BB, et al. Analysis of Fecal Sludges Reveals Common Enteric Pathogens in Urban Maputo, Mozambique. Environ Sci Technol Lett [Internet]. 2020 Oct 13 [cited 2020 Oct 14];acs.estlett.0c00610. Available from: https://pubs.acs.org/doi/10.1021/acs.estlett.0c00610

24. World Health Organization. Guidelines on Sanitation and Health [Internet]. Geneva; 2018. 1–220 p. Available from: http://apps.who.int/iris/bitstream/handle/10665/274939/9789241514705-eng.pdf?ua=1

25. Strande L, Ronteltap M, Brdjanovic D. Faecal Sludge Quantification, Characterisation and Treatment Objectives. In: Faecal Sludge Management: Systems Approach for Implementation and Operation. London, UK: IWA Publishing; 2014. p. 19–44.

26. Peal A, Evans B, Blackett I, Hawkins P, Heymans C. A Review of Fecal Sludge Management in 12 Cities: Annexure A.4 Maputo, Mozambique. Water Sanit Progr [Internet]. 2015;(June):563. Available from: http://www.susana.org/en/resources/library/details/2212

27. Norman G, Pedley S, Takkouche B. Effects of sewerage on diarrhoea and enteric infections: A systematic review and meta-analysis. Lancet Infect Dis [Internet]. 2010 Aug [cited 2020 May 13];10(8):536–44. Available from: http://www.ncbi.nlm.nih.gov/pubmed/20620115

28. Barreto ML, Genser B, Strina A, Teixeira MG, Assis AMO, Rego RF, et al. Effect of city-wide sanitation programme on reduction in rate of childhood diarrhoea in northeast Brazil: assessment by two cohort studies. Lancet. 2007 Nov 10;370(9599):1622–8.

29. Chiposa R, Holm RH, Munthali C, Chidya RCG, de los Reyes FL. Characterization of pit latrines to support the design and selection of emptying tools in peri-urban Mzuzu, Malawi. J Water Sanit Hyg Dev [Internet]. 2017 Mar 1 [cited 2020 Nov 12];7(1):151–5. Available from: http://iwaponline.com/washdev/article-pdf/7/1/151/386195/washdev0070151.pdf

30. Bourgault C, Shaw K, Dorea CC. Dominant decomposition pathways in pit latrines: a commentary. Water Sci Technol. 2019 Oct 1;80(7):1392–4.

31. Byrne A, Sindall R, Wang L, De los Reyes F, Buckley C. What happens inside a pour-flush pit? Insights from comprehensive characterization. In: Shaw RJ, editor. Local action with international cooperation to improve and sustain water, sanitation and hygiene (WASH) services: Proceedings of the 40th WEDC International Conference [Internet]. Loughborough, UK: WEDC, Loughborough University; 2017. Available from: https://repository.lboro.ac.uk/articles/What_happens_inside_a_pour-flush_pit_Insights_from_comprehensive_characterization/9589328

32. Changara M, Sanyika W, Bangira C, Misi S. Physico-chemical properties and bacterial community structure dynamics during the mesophilic anaerobic digestion of pit latrine faecal sludge. Water SA. 2019;45(3):338–48.

33. Letah Nzouebet WA, Noumsi IMK, Rechenburg A. Prevalence and diversity of intestinal helminth eggs in pit latrine sludge of a tropical urban area. J Water Sanit Hyg Dev. 2016 Dec 1;6(4):622–30.

34. Nabateesa S, Zziwa A, Kabenge I, Kambugu R, Wanyama J, Komakech AJ. Occurrence and survival of pathogens at different sludge depths in unlined pit latrines in Kampala slums. Water SA [Internet]. 2017 Nov 6;43(4):638. Available from: https://www.ajol.info/index.php/wsa/article/view/162569

35. Madhi SA, Cunliffe NA, Steele D, Witte D, Kirsten M, Louw C, et al. Effect of Human Rotavirus Vaccine on Severe Diarrhea in African Infants. N Engl J Med [Internet]. 2010 Jan 28;362(4):289–98. Available from: http://www.nejm.org/doi/abs/10.1056/NEJMoa0904797

36. Webster JP, Molyneux DH, Hotez PJ, Fenwick A. The contribution of mass drug administration to global health: past, present and future. Philos Trans R Soc B Biol Sci [Internet]. 2014 Jun 19;369(1645):20130434. Available from: https://royalsocietypublishing.org/doi/10.1098/rstb.2013.0434

37. Korich DG, Mead JR, Madore MS, Sinclair NA, Sterling CR. Effects of ozone, chlorine dioxide, chlorine, and monochloramine on Cryptosporidium parvum oocyst viability. Appl Environ Microbiol [Internet]. 1990;56(5):1423–8. Available from: https://aem.asm.org/content/56/5/1423

38. Farling S, Rogers T, Knee JS, Tilley EA, Brown J, Deshusses MA. Bioaerosol emissions

associated with pit latrine emptying operations. Sci Total Environ [Internet]. 2019 Jan 15;648:1082–6. Available from: http://www.ncbi.nlm.nih.gov/pubmed/30340254

39. Kodani M, Winchell JM. Engineered combined-positive-control template for real-time reverse transcription-PCR in multiple-pathogen-detection assays. J Clin Microbiol. 2012 Mar;50(3):1057–60.

40. Jaccard P. Étude comparative de la distribution florale dans une portion des Alpes et des Jura. Bull la Société vaudoise des Sci Nat. 1901;37:547–79.

41. Bates D, Machler M, Bolker B, Walker S. Fitting Linear Mixed-Effects Models Using {lme4}. J Stat Softw. 2015;67:1–48.

42. Fan Y-M, Oikarinen S, Lehto K-M, Nurminen N, Juuti R, Mangani C, et al. High prevalence of selected viruses and parasites and their predictors in Malawian children. Epidemiol Infect [Internet]. 2019 Feb 22 [cited 2020 Oct 20];147:e90. Available from: https://doi.org/10.1017/S0950268819000025

43. Mason J, Iturriza-Gomara M, O'Brien SJ, Ngwira BM, Dove W, Maiden MCJ, et al. Campylobacter Infection in Children in Malawi Is Common and Is Frequently Associated with Enteric Virus Co-Infections. Hold GL, editor. PLoS One [Internet]. 2013 Mar 26 [cited 2020 Sep 15];8(3):e59663. Available from: https://dx.plos.org/10.1371/journal.pone.0059663

44. Iturriza-Gómara M, Jere KC, Hungerford D, Bar-Zeev N, Shioda K, Kanjerwa O, et al. Etiology of Diarrhea among Hospitalized Children in Blantyre, Malawi, following Rotavirus Vaccine Introduction: A Case-Control Study. J Infect Dis [Internet]. 2019 Jun 19 [cited 2020 Oct 20];220(2):213–8. Available from: https://academic.oup.com/jid/article/220/2/213/5366554

45. Lehto K, Fan Y, Oikarinen S, Nurminen N, Hallamaa L, Juuti R, et al. Presence of *Giardia lamblia* in stools of six- to 18- month old asymptomatic Malawians is associated with children's growth failure. Acta Paediatr [Internet]. 2019 Oct 27 [cited 2020 Nov 4];108(10):1833–40. Available from: https://onlinelibrary.wiley.com/doi/abs/10.1111/apa.14832

46. Phiri KS. The prevalence, intensity and ecological determinants of helminth infection among children in an urban and rural community in Southern Malawi. Malawi Med J [Internet]. 2001 Sep;13(3):22–6. Available from: http://www.ncbi.nlm.nih.gov/pubmed/27528898

47. Feasey NA, Archer BN, Heyderman RS, Sooka A, Dennis B, Gordon MA, et al. Typhoid fever and invasive nontyphoid salmonellosis, Malawi and South Africa. Emerg Infect Dis [Internet]. 2010 [cited 2020 Sep 15];16(9):1448–51. Available from: /pmc/articles/PMC3294972/?report=abstract

48. Hosseinipour MC, Napravnik S, Joaki G, Gama S, Mbeye N, Banda B, et al. HIV and Parasitic Infection and the Effect of Treatment among Adult Outpatients in Malawi. J Infect Dis [Internet]. 2007 May 1 [cited 2020 Sep 15];195(9):1278–82. Available from: https://academic.oup.com/jid/article-lookup/doi/10.1086/513274

49. Hanevik K, Hausken T, Morken H, Strand EA, Mørch K, Coll P, et al. Persisting symptoms and duodenal inflammation related to Giardia duodenalis infection. J Infect [Internet]. 2007 [cited 2020 May 27];55:524–30. Available from: www.elsevierhealth.com/journals/jinf

50. Levine MM, DuPont HL, Khodabandelou M, Hornick RB. Long-Term Shigella-Carrier State. N Engl J Med [Internet]. 1973 May 31;288(22):1169–71. Available from: http://www.nejm.org/doi/abs/10.1056/NEJM197305312882207

51. Stelmach R, Clasen T. Household Water Quantity and Health: A Systematic Review. Int J Environ

Res Public Health [Internet]. 2015 May 28;12(6):5954–74. Available from: http://www.mdpi.com/1660-4601/12/6/5954

52. Rachwal PA, Rose HL, Cox V, Lukaszewski RA, Murch AL, Weller SA. The potential of TaqMan array cards for detection of multiple biological agents by real-time PCR. PLoS One. 2012;7(4).

53. Liu J, Gratz J, Amour C, Kibiki G, Becker S, Janaki L, et al. A laboratory-developed taqman array card for simultaneous detection of 19 enteropathogens. J Clin Microbiol. 2013;51(2):472–80.

54. Liu J, Gratz J, Amour C, Nshama R, Walongo T, Maro A, et al. Optimization of Quantitative PCR Methods for Enteropathogen Detection. Chan KH, editor. PLoS One [Internet]. 2016 Jun 23;11(6):e0158199. Available from: http://dx.plos.org/10.1371/journal.pone.0158199

55. Liu J, Platts-Mills JA, Juma J, Kabir F, Nkeze J, Okoi C, et al. Use of quantitative molecular diagnostic methods to identify causes of diarrhoea in children: a reanalysis of the GEMS case-control study. Lancet (London, England) [Internet]. 2016 Sep 24 [cited 2019 Mar 30];388(10051):1291–301. Available from: http://www.ncbi.nlm.nih.gov/pubmed/27673470

Click here to access/download
Supporting Information
Supporting Information.docx
