# Supplementary material for: Impact of sampling depth on pathogen detection in pit latrines
Source: PLoS Negl Trop Dis. 2021 Mar 2;15(3):e0009176. doi: 10.1371/journal.pntd.0009176 (PMC7954291; doi:10.1371/journal.pntd.0009176)
Supplement: Attachment — Submitted filename: PNTD-D-20-02024_KaderNdiaye_Review.pdf [file pntd.0009176.s011.pdf]

The main objective of your study was to to evaluate the spatial distribution of enteric pathogens inside pit latrines.Your finally suggested a single top, or a composite of top samples, for collecting specimens.

1- ***What risk is targeted by the collection in WBE purpose ?***
   - ***a)*** Risk to Groundwater via Subsurface Transport ?
   - ***b)*** Risk Incurred With Exposure From Pit Toilet ?
   - ***c)*** Risk Incurred With Emptying Pit

2- Did you assess environmental factors affecting Pathogen Reduction in Pit Toilets and Surrounding Soil in your analysis ?

The faecal sludge can be divided into four different layers (Nwaneri et al. 2008)

- Rapid aerobic degradation
- Rate limited aerobic degradation
- Anaerobic degradation
- Non degradable materials

Factors affecting pathogen reduction and removal in the **upper three zones** of the pit can be categorized as ***environmental*** *(*oxygen availability (+++), temperature, moisture content, storage time, pH, and volatile fatty acid content with microbicidal effects at low pH values) and *microbiological* (90% of microorganisms from the human gut are anaerobic; only 10% are aerobes or facultative anaerobes.Thus, the majority of microorganisms would have a survival advantage under the environmental conditions found in the lower anaerobic layers of the pit)

3- Why didn't you include bacterial indicators such as E. coli, total coliforms, or faecal coliforms to detect the presence of potential bacterial pathogens in groundwater near latrines because they are easier to monitor than pathogens and because both are cotransported from latrines ?
