# Supplementary material for: Impact of sampling depth on pathogen detection in pit latrines
Source: PLoS Negl Trop Dis. 2021 Mar 2;15(3):e0009176. doi: 10.1371/journal.pntd.0009176 (PMC7954291; doi:10.1371/journal.pntd.0009176)
Supplement: Attachment — Submitted filename: Response to Reviewers_Capone et al_January 2020.docx [file pntd.0009176.s012.docx]

Capone *et al*.
Manuscript Title: Impact of sampling depth on enteric pathogen detection in pit latrines

Thank you to all three reviewers for your time and feedback. The line numbers referenced below all refer to the “tracked changes” version of the manuscript.

| Reviewer 1 Comment | Author Response | Action Taken |
| --- | --- | --- |
| 1. Only a suggestion, instead of household population and whether the pit latrine was shared with other households, authors could adjusted by the number of members using the latrine (if available). | We agree that adjusting for the total population using the latrine would be desired over the household population and whether the latrine was shared. However, we only collected the household population and a binary variable for whether the latrine was shared with other households, so we can only adjust for these variables in our regression analysis. | None. |
| 2. Table 1, In my opinion it is a bit confusing the total number of latrines sampled, the number of latrines tat were contaminated and the number of parasites detected. I would clarify that. | Agreed. | Revised Table 1 to specify the number of samples for each column, substituted the IQR with the values for the 25^th^ and 75^th^ percentiles, and included additional text below the table. |

| Reviewer 2 Comment | Author Response | Action Taken |
| --- | --- | --- |
| 1. Line 139 - 145: The SI units should be standardise across the document. | Agreed | Revised throughout to specify the SI units. See lines 162-188 |
| 2. Line 148 - MS2 should be clearly defined | Agreed | We defined MS2. Line 180 |
| 3. Line 196 - The total in column 3 doesn't seem to add up | Thanks for noticing this. The count of pathogens detected were not normally distributed, so reporting the median and interquartile range is more appropriate than reporting the mean and standard deviation. Since we are reporting the median, and not the mean, the median at the bottom of column three does not need to equal the sum of the three values above. | None. Also see Reviewer 1’s Comment #2 |
| 4. Line 206 & 213 - I can't seem to understand the inter and intra latrine section. How are they defined? | Agreed. | We revised these titles to more clearly describe their respective sections. We also revised the language we used to describe pit latrine depth throughout to be more precise (e.g. surface, mid-point, and maximum depth instead of top, middle, and bottom).  Lines 242 and 249 |

| Reviewer 3 Comment | Author Response | Action Taken |
| --- | --- | --- |
| 1. What risk is targeted by the collection in WBE purpose ?     a) Risk to Groundwater via Subsurface Transport ?  b) Risk Incurred With Exposure From Pit Toilet ?  c) Risk Incurred With Emptying Pit | We agree that assessing infection risks from exposure to fecal sludges, or enteric pathogens in environmental matrices that may become contaminated by fecal sludges, is an important research question. All three of these scenarios are important transmission routes that should be considered. Quantitative Microbial Risk Assessment (QMRA) is one such tool to estimate these risks. While such an approach was outside the scope of this study, this is an important area of future research. | We revised the introduction to provide further justification for a WBE approach in LMIC settings unserved by sewers.  Lines 82-116 |
| 2. Did you assess environmental factors affecting Pathogen Reduction in Pit Toilets and Surrounding Soil in your analysis ?   The faecal sludge can be divided into four different layers (Nwaneri et al. 2008)   Rapid aerobic degradation   Rate limited aerobic degradation   Anaerobic degradation   Non degradable materials | Thank you for sharing this reference. Our analysis was cross-sectional and did not assess pathogen decay in fecal sludges or the surrounding environments. We agree this is an important area of future work and mention this on lines 345-348. | None |
| 3. Why didn't you include bacterial indicators such as E. coli, total coliforms, or faecal coliforms to detect the presence of potential bacterial pathogens in groundwater near latrines because they are easier to monitor than pathogens and because both are cotransported from latrines ? | We agree that measuring fecal indicator bacteria can be useful proxies for enteric pathogens in some environmental matrices. However, fecal indicator bacteria such as *E. coli* and fecal coliforms are commensal. Since waste-based epidemiology requires data on etiological agents we focused our study on the molecular detection of common enteric pathogens and not fecal indicator bacteria.  We agree that future work exploring the spread of pathogens from fecal sludges to groundwater and other environmental matrices should consider fecal indicator bacteria and pathogen viability, which was outside the scope of this study, but is an important consideration. | None |
